# Crosstalk Between Kappa Opioid and Dopamine Systems in Compulsive Behaviors

**DOI:** 10.3389/fphar.2020.00057

**Published:** 2020-02-18

**Authors:** Angélica del Pilar Escobar, José Patricio Casanova, María Estela Andrés, José Antonio Fuentealba

**Affiliations:** ^1^Centro Interdisciplinario de Neurociencias de Valparaíso, Faculty of Sciences, Universidad de Valparaíso, Valparaíso, Chile; ^2^Departamento de Neurociencia, Facultad de Medicina, Universidad de Chile, Santiago, Chile; ^3^Núcleo Milenio NUMIND Biology of Neuropsychiatric Disorders, Universidad de Valparaíso, Valparaíso, Chile; ^4^Department of Cellular and Molecular Biology, Faculty of Biological Sciences, Pontificia Universidad Católica de Chile, Santiago, Chile; ^5^Department of Pharmacy and Interdisciplinary Center of Neuroscience, Faculty of Chemistry, Pontificia Universidad Católica de Chile, Santiago, Chile

**Keywords:** kappa opioid receptor, dopamine, compulsivity, amphetamine, quinpirole, locomotor sensitization

## Abstract

The strength of goal-oriented behaviors is regulated by midbrain dopamine neurons. Dysfunctions of dopaminergic circuits are observed in drug addiction and obsessive-compulsive disorder. Compulsive behavior is a feature that both disorders share, which is associated to a heightened dopamine neurotransmission. The activity of midbrain dopamine neurons is principally regulated by the homeostatic action of dopamine through D2 receptors (D2R) that decrease the firing of neurons as well as dopamine synthesis and release. Dopamine transmission is also regulated by heterologous neurotransmitter systems such as the kappa opioid system, among others. Much of our current knowledge of the kappa opioid system and its influence on dopamine transmission comes from preclinical animal models of brain diseases. In 1988, using cerebral microdialysis, it was shown that the acute activation of the Kappa Opioid Receptors (KOR) decreases synaptic levels of dopamine in the striatum. This inhibitory effect of KOR opposes to the facilitating influence of drugs of abuse on dopamine release, leading to the proposition of the use of KOR agonists as pharmacological therapy for compulsive drug intake. Surprisingly, 30 years later, KOR antagonists are instead proposed to treat drug addiction. What may have happened during these years that generated this drastic change of paradigm? The collected evidence suggested that the effect of KOR on synaptic dopamine levels is complex, depending on the frequency of KOR activation and timing with other incoming stimuli to dopamine neurons, as well as sex and species differences. Conversely to its acute effect, chronic KOR activation seems to facilitate dopamine neurotransmission and dopamine-mediated behaviors. The opposing actions exerted by acute versus chronic KOR activation have been associated with an initial aversive and a delayed rewarding effect, during the exposure to drugs of abuse. Compulsive behaviors induced by repeated activation of D2R are also potentiated by the sustained co-activation of KOR, which correlates with decreased synaptic levels of dopamine and sensitized D2R. Thus, the time-dependent activation of KOR impacts directly on dopamine levels affecting the tuning of motivated behaviors. This review analyzes the contribution of the kappa opioid system to the dopaminergic correlates of compulsive behaviors.

## Introduction

### Dopaminergic System in Compulsive Behaviors

Compulsion is the impossibility of self-stopping to execute a habitual action with known outcome, despite adverse consequences (Robbins et al., 2012). Compulsive behaviors are hallmarks of obsessive-compulsive disorder (OCD) and drug addiction, among other psychiatric diseases. Checking behavior is very common in obsessive-compulsive spectrum disorders being characterized by the constant repetition of a certain routine, in a stereotyped or ritualistic way (Williams et al., 2013). A wide range of normal behaviors (e.g., checking, cleaning, hands washing, etc.) can turn into compulsive in OCD patients and in general, arises in response to obsessive and distressing thoughts inducing anxiety. Similarly, seeking and consuming drugs of abuse become compulsive in drug addicts As in OCD, anxiety plays a key role triggering compulsive drug consumption in experienced drug abusers. The same impairments in reward and punishment processing are observed in both conditions (Figee et al., 2016), which has led some authors to discuss OCD as a behavioral addiction (Holden, 2001).

One possible mechanism leading to compulsive behavior is framed within the incentive-sensitization theory of addiction which is that an amplified motivation (“wanting”) for the drug develops during addiction without developing an amplified pleasurable (“liking”) effect (Berridge et al., 1989; Berridge and Robinson, 2016). Enduring sensitization of the reward/motivation circuit is involved in the induction of incentive-sensitization associated to drug seeking. The reward/motivation circuit is composed of midbrain dopamine neurons of the *substantia nigra* (SN) and ventral tegmental area (VTA), which target the dorsal and ventral tiers of the striatum, respectively. Dopamine neurons that project to the ventral striatum or nucleus accumbens (NAc) have been traditionally related to goal-oriented behaviors, whereas dopamine neurons that project to the dorsal striatum have been associated with habits acquisition (Everitt and Robbins, 2005; Wise, 2009; Yager et al., 2015; Volkow et al., 2017).

Sensitization of the reward/motivation circuit is observed in rodents as the gradual increase in locomotor activity induced by repeated administration of a potentially addictive drug fixed dose (Pierce and Kalivas, 1997; Robinson and Berridge, 2001). Locomotor sensitization is an endurable phenomenon as it is observable after weeks, months and even a year after drug withdrawal (Robinson and Berridge, 1993). It was early suggested that sensitization of the reward/motivation circuit contributes to the compulsive drug seeking (Robinson and Berridge, 1993). Accordingly, locomotor sensitization facilitates self-administration cocaine seeking reinstatement (De Vries et al., 2002). Moreover, rats with extended access to cocaine self-administration show greater locomotor response to cocaine than rats with limited access (Ferrario et al., 2005). In addition, the neurochemical changes underlying locomotor sensitization to psychostimulants are also observed in compulsive drug seeking (Steketee and Kalivas, 2011; Giuliano et al., 2019). These data support the early proposed correspondence between locomotor sensitization and compulsive drug seeking observed in humans (Robinson and Berridge, 1993; Vanderschuren and Kalivas, 2000). Mechanistically, repeated administration of drugs of abuse sensitizes mesolimbic dopamine circuits increasing dopaminergic neurotransmission. Psychostimulants, like cocaine or amphetamines, that block the plasma membrane dopamine transporter (DAT), induce a large increase of dopamine in the synaptic space in the striatum and NAc, thus activating locomotion (Steketee and Kalivas, 2011). As in drug addiction, sensitization of the dopamine reward/motivation circuit contribute to compulsive behaviors seen in OCD. Indeed, the repeated activation of dopamine D2 receptors (D2Rs) is enough to induce locomotor sensitization and checking behavior in both rats and mice (Szechtman et al., 1998; Szechtman et al., 1999; Sun et al., 2019). Repeated administration of quinpirole, a D2R/D3R agonist, is an accepted model of OCD as it recapitulates face validity, through an increment of compulsive checking and stereotyped behavior, predictive validity, as seen by a decrease of compulsive behaviors after chronic treatment with serotonin reuptake inhibitors (SRI) and construct validity as brain structures involved in this model are shared with those in the pathology (Stuchlik et al., 2016; Szechtman et al., 2017). In summary, repeated activation of dopamine transmission, either by pre-synaptic (dopamine release) or post-synaptic (activation of D2R) mechanisms lead to locomotor sensitization and compulsive behaviors.

The kappa opioid system is one of the most preponderant systems controlling dopamine transmission in the reward/motivation circuit. Evidence shows that kappa-opioid transmission opposes to the effects of dopamine; the acute activation of kappa opioid receptors (KORs) counteracts the locomotor activity induced by psychostimulants (Gray et al., 1999). Conversely, repeated KOR activation maintains and enhances compulsive and habitual drug seeking (Koob, 2013). Consumption of drugs of abuse induce a homeostatic enhanced kappa opioid transmission, probably contributing to the negative emotional states of dysphoria (Koob, 2013) triggering compulsive drug use (Chavkin and Koob, 2016). In fact, the blockade of KOR prevented stress- but not drug-induced reinstatement of nicotine (Jackson et al., 2013), cocaine (Beardsley et al., 2005) and ethanol (Sperling et al., 2010). In line with this finding, KOR blockade reverts dopaminergic changes in the dorsolateral striatum of amphetamine sensitized rats, without modifying their enhanced locomotor response to the drug (Azocar et al., 2019). Thus, KOR system seems to enhance negative reinforcement increasing drug-value. In OCD, negative reinforcement is triggered by obsessions, which strengthen a given compulsion in order to avoid that obsession. Although it has not been directly tested, negative reinforcing could play a role on quinpirole sensitization. Indeed, D2R are involved in the generation of negative reinforcement. For example, place avoidance to a morphine- withdrawal-paired area was not developed in mice lacking the long isoform of D2R (Smith et al., 2002) and repeated quinpirole treatment during abstinence period reinstates cocaine and heroin seeking in an auto-administration paradigm, an effect related to sensitized locomotion to quinpirole (De Vries et al., 2002), suggesting shared mechanisms between psychostimulant and quinpirole-induced sensitization. Moreover, the introduction of the home cage, but not a novel cage, to the open-field arena reduces locomotor sensitization and compulsive checking behavior (Szechtman et al., 2001), indicating that safety/familiar cues might compete with negative environmental cues that favor sensitization. Similarly to psychostimulant-induced sensitization, the repeated activation of KOR facilitates locomotor sensitization (Escobar et al., 2017) and compulsive checking behavior (Perreault et al., 2007) induced by repeated administration of quinpirole. Whether this potentiating effect is a consequence of enhanced negative reinforcement remains to be elucidated.

The thorough analysis carried out recently shows that the effect of the kappa-opioid system on dopaminergic transmission is complex: it depends on the dopamine pathway involved (Margolis et al., 2006; Margolis et al., 2008), and on the timing between the activation of the KOR receptor and the activation of the dopamine receptor (Chartoff et al., 2016). Consistent with this complexity, the potential therapeutic use of KOR ligands has been widely discussed. It has been proposed that KOR agonist may be clinically useful during the drug use phase, attenuating the drug induced hyperdopaminergia (Shippenberg et al., 2007). On the other hand, a KOR antagonist may be useful in treating withdrawal syndrome induced by an increase in dynorphin expression after repeated drug consumption (Wee and Koob, 2010). Accordingly, it has been proposed that KOR partial agonist (Béguin et al., 2012) could be a therapeutic option to treat both the compulsive drug intake and withdrawal symptoms in addicted individuals (Chartoff et al., 2016; Callaghan et al., 2018). In this review, we analyze the time/context-dependent modulation of dopaminergic correlates of behavioral sensitization and compulsivity.

### Anatomical and Functional Crosstalk Between Kappa Opioid and Dopaminergic Systems in Striatal and Midbrain Regions

#### Striatal Regions

KORs are Gi/o protein-coupled receptors highly expressed in the midbrain dopamine system (Mansour et al., 1996). These receptors belong to the family of opioid receptors composed by mu (MOR), delta (DOR) and kappa (KORs). The endogenous agonists for these receptors are endorphins, enkephalin and dynorphin, respectively. In the striatum, dynorphin is synthetized by dopamine D1receptor (D1R)-containing medium-sized neurons (MSNs) that have recurrent axons activating KORs from the same nuclei (Mansour et al., 1995). Electron microscopy images of rat NAc shows that KORs are found predominantly in DAT-containing presynaptic structures while a minor proportion of KORs localizes on dendrites in apposition to DAT (Svingos et al., 2001; Kivell et al., 2014). Immunofluorescent studies characterizing presynaptic-synaptosomal preparations from NAc show that KORs and D2Rs preferentially coexist in synaptosomes containing the dopamine synthetizing enzyme, tyrosine hydroxylase (TH) (Escobar et al., 2017). Moreover, KORs are abundant in cell bodies of the NAc and striatum, and colocalize with D2Rs in a cell subpopulation (Escobar et al., 2017). With genetic and molecular insights, it has been suggested that a 20% of total KOR binding in the striatum is observed in DA terminals (Van't Veer et al., 2013). Moreover, Tejeda et al. (2017) showed that both D1R and D2R MSNs express KOR with a higher preference for D1R containing MSNs (Tejeda et al., 2017). This anatomical data indicates that KORs are present pre and postsynaptically, regulating dopamine neurotransmission in the reward/motivation circuit.

Several experimental approaches show that the activation of KORs inhibits dopamine release. The acute activation of KORs by a systemic injection or the local infusion of agonists decreases the extracellular levels of dopamine in the NAc (Di Chiara and Imperato, 1988; Spanagel et al., 1992; Fuentealba et al., 2006) and dorsal striatum (Gehrke et al., 2008). Supporting a tonic inhibitory action of KORs over dopamine neurotransmission, the direct infusion of the long-lasting and selective KOR antagonist nor-binaltorphimine (nor-BNI) (Broadbear et al., 1994) increases basal levels of dopamine in the NAc (Spanagel et al., 1992) and dopamine release in the dorsal striatum (Azocar et al., 2019). Final evidence of KOR tonic inhibition of dopamine was shown in KOR knockout mice, which displayed increased extracellular levels of dopamine in the striatum and NAc (Chefer et al., 2005). The mechanisms responsible for KOR inhibition of dopamine release are not completely elucidated. However, it is well known that the activation of KORs leads to the increase of K + and decrease of Ca2+ conductances, thus inducing cell hyperpolarization and blockade of vesicular neurotransmitter release (Bruchas and Chavkin, 2010; Margolis and Karkhanis, 2019).

Additionally*, in vitro* and *in vivo* functional data suggests that KORs modify dopamine extracellular levels by modulating the activity of DAT. For instance, the activation of KORs in EM4 cells that co-express KORs and DAT, lead to an increased uptake of dopamine measured by voltammetry (Kivell et al., 2014). An *ex vivo* analysis also using voltammetry in disaggregated tissues, showed that a systemic injection of KOR agonist U-69593 increased dopamine uptake in the NAc (Thompson et al., 2000). A similar recent article shows that nor-BNI blocks the increase of dopamine uptake in the ventral and dorsal striatum, induced by an acute systemic injection of MP1104, a mixed Kappa/Delta opioid receptor agonist (Atigari et al., 2019). Nevertheless, the effect of KOR activation on dopamine uptake has yet not been not fully elucidated. The systemic administration of the KOR partial agonist nalmefene decreased striatal dopamine uptake dose dependently, quantified by fast scan cyclic voltammetry (FSCV) (Rose et al., 2016). Using a no-net flux microdialysis in adult male rats, blocking of KOR was accompanied by an increase in extraction fraction (Ed), which is an indirect measure of dopamine uptake (Chefer et al., 2006; Azocar et al., 2019), suggesting that tonic activation of KOR exerts an inhibitory control on DAT activity (dopamine uptake). These results highlight the complex role of endogenous KOR activity on dopamine uptake to control dopamine extracellular levels. Higher temporal resolution approaches such as FSCV have failed to show an effect of KOR on dopamine uptake (Ebner et al., 2010; Ehrich et al., 2015; Hoffman et al., 2016), suggesting that KOR enhancing DAT activity in striatal regions needs an incubation period., KOR-mediated enhancement of DAT activity could be explained by an increase in the number of DAT on cell membranes induced by KOR activation, as reported in striatal synaptosomes and cell lines (Kivell et al, 2014).

#### Midbrain Regions

Autoradiographic assays performed in the rat midbrain show significant binding for KORs on the rostrocaudal axis of the SN and VTA (Speciale et al., 1993). On the other hand, electron microscopy data show that dynorphin-containing terminals synapse directly on TH positive dendrites in the SN and the VTA (Sesack and Pickel, 1992), suggesting that KORs localize in somatodendritic compartments of dopamine neurons. Striatal D1R-containing MSNs are one of the dynorphin inputs to midbrain dopamine neurons. Interestingly, blockage of KORs does not modify the inhibitory effect of D1R-MSNs to VTA dopamine neurons, indicating that this inhibition is mediated by GABA (Edwards et al, 2017). KORs modulate somatodendritic responses of dopamine midbrain neurons. Electrophysiological studies show that the activation of KORs in the VTA hyperpolarizes and decreases the spontaneus firing rate of dopamine neurons (Margolis et al., 2003). Consequently, the infusion of KOR agonists decreases somatodendritic dopamine efflux (Smith et al., 1992; Dalman and O'Malley, 1999). However, this inhibitory effect of KORs on dopamine neurons seems to be circuit dependent. The infusion of kappa-opioid agonists in the VTA decreases dopamine release in the medial prefrontal cortex (mPFC) (Margolis et al., 2006) but not in the NAc (Devine et al., 1993; Margolis et al, 2006). Moreover, Margolis et al. (2006) found that KORs inhibit VTA dopamine neurons that project to the mPFC and basolateral amygdala, but not those that project to the NAc. In that same year, Ford et al. (2006) showed that bath application of KOR agonists in mouse VTA slices induced a higher outward current in dopamine neurons that project to the NAc compared to those that project to the basolateral amygdala, indicating that KORs exert a greater inhibition of dopamine neurons that project to the NAc than to the amygdala. Furthermore, the activation of KOR decreases the amplitude of excitatory (Margolis et al., 2005) and inhibitory (Ford et al., 2007) postsynaptic currents into midbrain dopamine neurons. Differences between species and the complex efferents proyections of VTA to mPFC and NAc (Van Bockstaele and Pickel, 1995; Carr and Sesack, 2000) make it challenging to establish whether KORs inhibit selectively some of the neuronal dopamine populations in VTA. Nevertheless, the data summarized here indicates that KORs are in the soma and terminals of dopamine neurons, as well as in the inputs that regulate them, thus exquisitely positioned to control the synaptic activity of midbrain dopamine neurons.

### Role of KORs Controlling Dopamine Neurotransmission in Psychostimulants-Induced Sensitization and Compulsive Behaviors

Drug addiction is a process that involves initially impulsive drug seeking associated with their positive-reinforcing effects. On the other hand, compulsivity is a personality trait observable in drug addicts. Several neuroadaptations in dopaminergic pathways have been proposed to account for compulsive drug seeking and intake following repeated exposure to drugs of abuse (Everitt and Robbins, 2005; Koob and Volkow, 2016). One of the proposed hypotheses driving compulsive drug intake is the sensitization of its negative-reinforcing effects (Koob, 2013). The inhibitory control of kappa opioid system on dopamine release could contribute to the negative-reinforcing properties of drugs of abuse. However, the consequences of KOR activation on dopamine neurotransmission and compulsive drugs seeking seems to be complex and apparently contradictory. Indeed, dopamine release induced by amphetamine and cocaine is attenuated by concomitant administration of KOR agonists (Heidbreder and Shippenberg, 1994; Maisonneuve et al., 1994; Thompson et al., 2000) and even decrease cocaine self-administration (Negus et al., 1997). Moreover, KORs exert an inhibitory feedback on dopamine release of the mesolimbic pathway in response to the sustained activation of post-synaptic D1R as occurs with repeated exposure to psychostimulants (Cole et al., 1995; Nestler, 2001). Paradoxically, the activation of KORs can also facilitate dopamine release in the reward/motivation pathway (Fuentealba et al., 2006; Fuentealba et al., 2007) and psychostimulants consumption (Wee et al., 2009). Fuentealba et al. (2007) showed that after four days administering U69593, a KOR agonist, increased amphetamine induced dopamine release in the NAc. Recently, it was shown that blocking KORs reverses the changes in dopamine release and uptake in dorsal striatum that takes place during the locomotor sensitization induced by amphetamine (Azocar et al., 2019). Altogether, these data suggest that the activation of KORs might also contribute to positive-reinforcing properties of drug of abuse (Chartoff et al., 2016).

In addition, KORs activation also seem to contribute to compulsive drug seeking; KORs blockade reduces cocaine (Wee et al., 2009), heroin (Schlosburg et al., 2013) and methamphetamine (Whitfield et al., 2015) intake in rats with unlimited access to the drug (Wee et al, 2009). This effect is also evidenced in stress-induced drug seeking. For instance, the KOR knockout mice did not show cocaine place preference after forced swimming stress (McLaughlin et al., 2006a). The blocking of KORs attenuates the nicotine place preference induced by forced swim stress exposure (Smith et al., 2012). Interestingly, the blocking of KOR attenuates the cocaine and nicotine seeking induced by stress but did not affect seeking induced by a drug challenge (Beardsley et al., 2005; Jackson et al., 2013). This facilitator KOR effect induced by stress seems to be mediated by the reward/motivation circuit (Shippenberg et al., 2007; Wee and Koob, 2010). In an elegant study performed by Dr. Kauer and her group, it was shown that blocking KORs in the VTA, either previously or after an acute stress, inhibits the reinstaintment of cocaine-seeking, an effect associated to the rescue of long-term-potentiation of inhibitory synapses in dopamine neurons (Graziane et al., 2013; Polter et al., 2014).

The facilitation of psychostimulants intake exerted by KORs seems to depend on a time-window regarding drug exposure. The administration of the KOR agonist U50488 1 h before cocaine exposure potentiates both cocaine place preference and the relative dopamine release evoked by cocaine in the NAc, while the opposite effects are observed when given 15 min before (McLaughlin et al., 2006a; Ehrich et al., 2014). Using intracranial self-stimulation Chartoff et al. (2016) observed that the KOR agonist Salvinorin A, has an initial aversive and a delayed rewarding effect, accompanied by a decrease and an increase in stimulated dopamine release in the NAc, respectively. All together these data indicate a time-dependent effect of KOR activation on the rewarding properties of cocaine, and points to the stress-mediated KOR activation as a key player for the development of compulsive drug-seeking.

### Quinpirole-Induced Locomotor Sensitization and Compulsive Behavior

The facts that the dopamine system is involved in the generation of sensitization and compulsivity is strengthened by the behavior observed in rodents treated with the D2R agonist, quinpirole. Briefly, D2Rs are Gi coupled receptors widely expressed in the reward/motivation circuit; they are expressed somatodendritically and on axon terminals of dopamine neurons (Sesack et al., 1994), and its activation decreases dopamine extracellular levels (Imperato and Di Chiara, 1988). In the striatum, D2Rs are also located postsynaptically on medium spiny neurons (Sesack et al., 1994) and its activation inhibits the indirect pathway allowing locomotor activity.

Dr. Henry Szechtman began studying the effects of quinpirole on the behavior of rats ending the decade of 1980. Their initial findings showed that the acute administration of quinpirole has dose-dependent effect on locomotor activity. At low doses (0.03 mg/kg) it decreases locomotor activity, while at higher doses (>0.5 mg/kg), it increments. (Eilam and Szechtman, 1989). These effects are associated with the activation of high-affinity presynaptic D2Rs and low-affinity postsynaptic D2Rs, respectively (Usiello et al., 2000). Unexpectedly, the repeated (every other day) administration of quinpirole induces a gradual and sustained increase in locomotion, resembling the locomotor sensitization induced by psychostimulants (Szechtman et al., 1993; Szechtman et al., 1994). The locomotor sensitizing effect was shown to depend on D2Rs, since mice deficient for this receptor do not develop locomotor sensitization to quinpirole (Escobar et al., 2015).

At the beginning of the 90's, Szechtman and Eilam reported that along with locomotor sensitization, rats developed a stereotyped behavior, which is reinforced with each administration of quinpirole (Eilam and Szechtman, 1989; Szechtman et al., 1993). Today, quinpirole repeated administration is a validated model for OCD (Szechtman et al., 1999; Szechtman et al., 2001; Eilam and Szechtman, 2005; Stuchlik et al., 2016; Szechtman et al., 2017), based on the observation that the behavior of rats becomes increasingly structured and inflexible, reminiscent of the ritual behavior characteristic of compulsive checking behavior (Szechtman et al., 1998; Szechtman et al., 2017). Recent studies show that repeated quinpirole also induces compulsive behaviors in mice, such as compulsive checking (Sun et al., 2019), behavioral inflexibility and compulsive chewing (Asaoka et al., 2019), the latter reverted by D2Rs blockade in the striatum, further supporting that repeated D2Rs activation is needed to induce compulsive behaviors. Together the data points to a crucial role of D2Rs within the midbrain dopamine pathways to induce locomotor sensitization and compulsivity. Repeated quinpirole administration primes cocaine-induced stereotyped behavior (Thompson et al., 2010) and the locomotor effects of amphetamine (Cope et al., 2010), strengthening the idea that D2Rs activation underlie psychostimulant-induced sensitization and suggesting a shared mechanism between quinpirole and psychostimulants-induced sensitization. Interestingly, the sensitizing effect of repeated D2Rs activation seems to be stronger than that induced by psychostimulants, since every rat treated with quinpirole develop locomotor sensitization (Escobar et al., 2015), while around sixty percent of rats sensitize to amphetamine (Escobar et al., 2012; Casanova et al., 2013).

Behavioral sensitization induced by repeated activation of D2Rs is accompanied by adaptations in the reward/motivation circuit. Rats sensitized with quinpirole have lower dopaminergic tone in the NAc, observed as decreased basal (Koeltzow et al., 2003) and stimulated tonic and phasic dopamine release (Escobar et al., 2015), indicating decreased dopamine release capacity of dopamine midbrain circuit. Synaptic dopamine levels in the NAc are controlled by the activity of both, DAT and dopamine neurons activity (Goto and Grace, 2008), which *in vivo* consists of tonic and burst firing (Wilson et al., 1977; Grace and Bunney, 1980). Previous reports show that quinpirole-sensitized rats display a smaller number of dopamine neurons in tonic and burst firing in the VTA (Sesia et al., 2013). Together these data indicate that the decrease in dopamine release seen after quinpirole sensitization is a result of a decrease in the overall activity of dopamine neurons. The compulsive behavior and sensitized locomotor activity induced by the repeated treatment with quinpirole could be a consequence of sensitization of D2Rs, due to decreased dopaminergic tone in the NAc. Indeed, quinpirole-sensitized rats show an increase in the binding of dopamine D2R (Culver et al., 2008) and an increase in the affinity state of these receptors (Perreault et al., 2007), supporting this hypothesis.

### KOR-Dopamine Interactions in Quinpirole-Induced Compulsive Behaviors

Initial studies regarding the role of KOR in D2R-induced compulsive behaviors also came from Szechtman's lab. This group examined the concomitant administration of the KOR agonist U69593 with quinpirole on locomotor activity. Specifically, the authors administered subcutaneous injections to rats with a mixture U69593 and quinpirole, until 8 to 10 injections were completed. Contrary to the hypolocomotor effect of U69593 alone, hyperlocomotion was observed when administered concomitantly with low (presynaptic) and high (postsynaptic) doses of quinpirole. U69593 changed the hypolocomotor effect of a presynaptic dose of quinpirole to hyperlocomotion and enhanced the hyperlocomotor effect of a postsynaptic dose of quinpirole (Perreault et al., 2006). Co-activation of KORs also accelerated the induction of locomotor sensitization and potentiated the effect of D2Rs activation, since the maximum locomotion achieved by the double treatment duplicates the locomotor effect induced by quinpirole alone (Perreault et al., 2006; Escobar et al., 2017). Co-activation of KORs also accelerates the acquisition of compulsive checking behavior (Perreault et al., 2007). These potentiating effects of KORs on quinpirole-induced behaviors require KORs repeated activation. In fact, acute injection of the KOR agonist U69593 did not further modify the locomotor activity in rats sensitized with quinpirole (Escobar et al., 2017). The mechanism of KOR potentiating D2R-induced sensitization is unknown. One possibility is that the endogenous kappa opioid system itself is mediating D2R-dependent sensitization. However, this possibility was discarded by showing that pre-administration of norBNI did not modify locomotor sensitization to quinpirole, suggesting that dynorphin is not released downstream D2R activation (Escobar et al., 2017). This data does not rule out that dynorphin might have a role in sensitizing compulsive behaviors, for example, stress induces the release of dynorphin and activation of KORs which facilitates compulsive behaviors (McLaughlin et al., 2003; McLaughlin et al., 2006a; McLaughlin et al., 2006b).

The crosstalk between D2Rs and KORs is complex and it seems to depend on whether the activation of both receptors is coincident or temporally separated. Anatomical data indicate that the crosstalk between D2Rs and KORs can occur presynaptically in axons and soma of dopamine neurons, as well as postsynaptically in MSNs of the striatum. Although it does not rule out a role for KORs located on axons of other neurochemical systems, the anatomical data strongly points to a direct role of KORs regulating D2Rs. Either acute or repeated, the activation of KORs decreases the inhibitory D2Rs function on dopamine neurons. Electrophysiology studies showed that the acute activation of KOR in dopamine neurons of the VTA and SN inhibits D2R-mediated inhibitory postsynaptic current, an effect mediated by pre and postsynaptic mechanisms as KOR decreases dopamine release and dynorphin blocks the inhibitory effect of bath applied dopamine (Ford et al., 2007). Neurochemical studies showed that the repeated activation of KORs blocks D2R-induced inhibition of dopamine release in the NAc (Fuentealba et al., 2006). Moreover, coincident D2Rs and KORs acute activation decreases the inhibition of dopamine release in the NAc compared to the effect of each receptor alone (Escobar et al., 2017). Thus, presynaptic KORs do not act additively or in synergy with presynaptic D2Rs, conversely, KORs either inhibit or occlude D2R inhibitory effect. This mechanism could explain the locomotor activating effect of an acute dose of KOR agonists concomitant to a low dose of quinpirole (Perreault et al., 2006).

A recent study shows that KOR activation in the VTA mediates compulsive behavior measured as behavioral inhibition and marble burying (Abraham et al., 2017), reinforcing the idea that KORs activation is indeed a trigger for compulsivity. Data published by Margolis et al. (2006; 2008) indicate that KORs and D2Rs interaction should take place on dopamine neurons targeting the mPFC (Margolis et al., 2006; Margolis et al., 2008). Notwithstanding, Ford et al. (2006; 2007) found that KORs inhibition of D2R mediated IPSC takes place on dopamine neurons targeting the NAc (Ford et al., 2006; Ford et al., 2007). Together these data show that KOR interaction with D2R at the somatodendritic compartment of dopamine neurons could arise as a result of a crosstalk in the same dopamine neuron. Whether this happens in the mesolimbic or mesocortical projections is still controversial.

**Figure 1:**
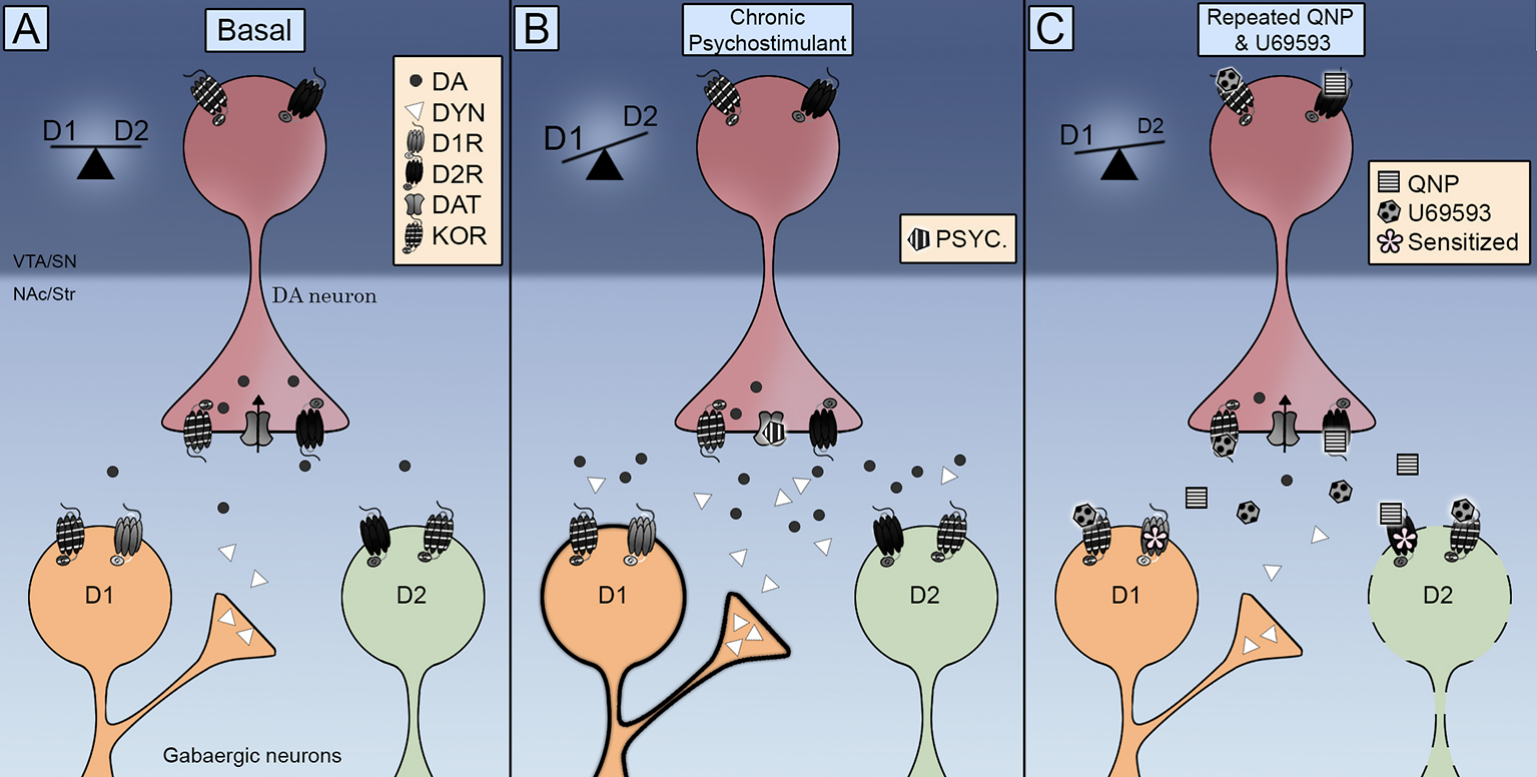
Integrative scheme of Kappa Opioid Receptors (KOR) control on direct (D1R) and indirect (D2R) striatal phatways. **(A)** KOR are located pre-sinaptically on dopamine terminals and post sinaptically in medium-sized neurons (MSNs). Its activation controls dopamine extracellular levels and its localization promotes the interaction with dopamine transporter (DAT) and dopamine D2 receptors. **(B)** The repeated exposure to a psychostimulant is accompanied by an increase in both dopamine extracellular levels and dynorphin. The activation of D1 and D2 receptors switch the balance to the D1R direct pathway promoting locomotor sensitization. **(C)** The co-administration of quinpirole and U69593 is accompanied by a decrease in dopamine extracellular levels. The concomitant activation of KOR and D2 receptors debilitates the D2 indirect pathway inducing compulsive behavior.

Remarkably, KOR was found in MSNs of the NAc (Escobar et al., 2017; Tejeda et al., 2017), thus indicating that the potentiation of D2R-induced compulsive behavior can also arise by direct actions on the target cells of dopamine neurons. In this regard, it is worth mentioning that repeated administration of U69593 increases the amount of D2Rs in the high affinity state (Perreault et al., 2007). Neurochemical data indicate that decreased dopamine extracellular levels is associated to D2Rs sensitization. KORs co-activation does not decrease further the extracellular levels of dopamine in the NAc already decreased by the repeated activation of D2Rs (Escobar et al., 2017), ruling out a role for presynaptic KORs accelerating or potentiating the sensitization of D2Rs in the NAc through this mechanism. Therefore, KORs trigger slow molecular mechanisms that further sensitize the neurochemical and behavioral effects of D2Rs, suggesting that the locomotion sensitization enhancement could be due to an adaptive postsynaptic rather than a presynaptic effect. In this regard repeated activation of KORs can trigger the inhibition of D2R indirect striatal pathway switching D1R/D2R balance to D1R inducing compulsivity (**Figure 1**).

### Sex Differences of KOR-Dopamine Interactions in Compulsive Behaviors

Clinical studies have shown sex differences in compulsive behavior including compulsive drug seeking. An earlier onset of OCD symptoms is observed in men compared with women (Mathis et al., 2011), with women showing more prevalence of contamination and cleaning symptoms (Labad et al., 2008). Regarding sex differences in drug addiction, clinical evidence indicates that while the use of drugs is more prevalent in men, women exhibit a faster progression than men into compulsive drug seeking (Hernandez-Avila et al., 2004; Fattore and Melis, 2016).

Lately, pre-clinical evidence has strongly highlighted the neurobiological bases underpinning the sex differences in drug abuse observed in clinical studies (Becker and Chartoff, 2019). Early observations using no-net flux microdialysis showed that dopamine extracellular concentration in the dorsal striatum varies during the estrous cycle with higher levels in proestrus and estrus compared with diestrus. Moreover, while ovariectomy decreases striatal dopamine extracellular concentration in female rats, the castration of male rats does not modify dopamine striatal extracellular concentration (Xiao and Becker, 1994), suggesting an important role of ovary hormones on dopamine activity. In addition, female hormones regulate the response to psychostimulants. Early *in vitro* experiments showed that estradiol plus progesterone restore amphetamine-induced dopamine release from striatal tissue obtained of ovariectomized female rats (Becker and Ramirez, 1981). More recently, fast scan cyclic voltammetry studies have shown that females exhibit greater electrically-stimulated dopamine release and uptake compared to males (Walker et al., 2000). These sex differences in dopamine neurotransmission can account for the higher cocaine and amphetamine seeking observed in females. (Roberts et al., 1989; Cox et al., 2013).

The regulation of KOR on dopamine extracellular levels also shows sex differences (Chartoff and Mavrikaki, 2015). Using intracranial self-stimulation and cyclic voltammetry, Conway et al. (2019) showed that the lower sensitivity to the acute anhedonic effect of a KOR agonist observed in female rats compared to male rats, is accompanied by an attenuated inhibition of stimulated dopamine release in the NAc (Conway et al., 2019). It has been suggested that estradiol contributes to the blunted inhibition of dopamine release observed in female rats after KOR activation (Abraham et al., 2018). While the crosstalk between KORs and dopamine signaling has been studied in males (Tejeda and Bonci, 2019), research on this interaction and its impact in the addiction process in females is lacking (Chartoff and Mavrikaki, 2015). In female rats, the acute administration of the KOR agonist U69593 attenuated cocaine-induced hyperlocomotion in both, control and ovariectomized rats. Interestingly, U69593 repeated administration attenuated cocaine-induced hyperlocomotion in an estradiol-dependent manner (Puig-Ramos et al., 2008). These data suggest that estradiol primes KOR actions in female rats, an effect that could be related to sex differences in stress response (Puig-Ramos et al., 2008). Whether in female rats the repeated activation of KORs facilitates striatal dopamine release as observed in male is an unanswered question.

Although a facilitation in psychostimulant induced dopamine release is observed in female compared to male rats, sex differences in the dopamine mechanisms underlying amphetamine locomotor sensitization have not been fully elucidated (Becker, 1999). The repeated exposure to amphetamine induces a greater locomotor activity in both, adolescent (Mathews and McCormick, 2007) and adult female rats (Milesi-Hallé et al., 2007), with female adolescent rats showing a more robust locomotor sensitization after repeated exposure to amphetamine. The neonatal activation of D2 receptor potentiated the amphetamine induced behavioral sensitization only in female rats (Brown et al., 2011). As mentioned before, it has been observed in male rats the repeated exposure to D2 agonist induces locomotor sensitization and compulsive-like behavior (Dvorkin et al., 2006). Moreover, the co-activation of KOR potentiates the locomotor sensitization induced by repeated exposure to quinpirole, facilitating the inhibitory control of D2 receptors on DA release in the NAc (Escobar et al., 2017). Sex differences such as the observed lower sensitivity to the inhibitory effect of KOR on dopamine release in females (Conway et al., 2019) may account for a differential contribution of KOR on compulsive drug seeking.

## Conclusions

How do KORs modulate dopamine signaling to elaborate motivated behaviors and when does it result in a sensitized compulsive behavior? Anatomical data shows that KORs are exquisitely positioned to control the synaptic activity of midbrain dopamine neurons. Functional data indicate that KORs control DAT and D2R functioning as well as dopamine neurons firing rate. Initial evidence showing that the acute activation of KORs decreases dopamine release induced by drugs of abuse has been complemented with data indicating that the repeated activation of KOR facilitates dopamine release and compulsive drug-seeking. Dopamine signaling balance direct and indirect output pathways from striatal areas (**Figure 1A**). Either chronic stimulation with psychostimulants that increases dopamine release activating both D1R and D2R (**Figure 1B**) or quinpirole that activate only D2R (**Figure 1C**) results in locomotor sensitization and compulsive behaviors by a debilitated D2R indirect pathway, thus switching the balance to the D1R direct pathway. KOR transmission is enhanced during chronic psychostimulant intake by the increase of dynorphin in striatal D1 neurons (**Figure 1B**). An enhanced KOR transmission is emulated in the pharmacological model of OCD by administering U69593. This concomitant KOR activation further debilitates the D2 indirect pathway (**Figure 1C**). Future research should be carried out to fully elucidate the consequences of KOR activation on the DAT activity, understand the role of endogeous KOR system in the quinpirole induced compulsivity and determine the contribution of KOR system to the sex diffences observed in compulsive behaviors.

## Author Contributions

AE, MA, and JF contributed to the conception of the manuscript. AE and JF wrote the first draft of the manuscript with input from MA. MA and JC contributed to the critical review and editing of the manuscript. All the authors approved it for publication.

## Funding

The work of the authors cited in this review has been supported by FONDECYT grant numbers: 1110352 and 1150200 to MA; 1141088 to JF; DIPOG grant 391340281 to JF; FONDECYT Postdoctoral fellow 3170497 to JC and 3190843 to AE.

## Conflict of Interest

The authors declare that the research was conducted in the absence of any commercial or financial relationships that could be construed as a potential conflict of interest.

The handling editor is currently organizing a Research Topic with one of the authors JF, and confirms the absence of any other collaboration.
